# Effect of Magnetic Resonance Imaging at 1.5 T and 3 T on Temperature and Bond Strength of Orthodontic Bands with Welded Tubes: An In Vitro Study

**DOI:** 10.3390/ma16020651

**Published:** 2023-01-09

**Authors:** Maria Francesca Sfondrini, Simone Gallo, Maurizio Pascadopoli, Cinzia Rizzi, Andrea Boldrini, Simone Santagostini, Luca Anemoni, Maria Sole Prevedoni Gorone, Lorenzo Preda, Paola Gandini, Andrea Scribante

**Affiliations:** 1 Unit of Orthodontics and Pediatric Dentistry, Section of Dentistry, Department of Clinical, Surgical, Diagnostic and Pediatric Sciences, University of Pavia, 27100 Pavia, Italy; 2 Department of Radiology, Fondazione IRCCS Policlinico San Matteo, University of Pavia, 27100 Pavia, Italy; 3 National Center for Oncological Hadrontherapy (CNAO), 27100 Pavia, Italy; 4 Department of Clinical, Surgical, Diagnostic and Pediatric Sciences, University of Pavia, 27100 Pavia, Italy

**Keywords:** composite cement, glass ionomer cement, molar bands

## Abstract

Magnetic resonance imaging (MRI) is a widely used diagnostic technique. Patients wearing orthodontic devices are often requested to remove their appliances before an MRI exam, even when the exam involves anatomical areas far from the head, in order to prevent the heating and detachment of the appliances. The present report aims to evaluate changes in temperature and adhesive forces of molar bands after MRI at two different strength outputs. Sixty stainless steel molar bands were bonded on permanent human upper molars using two different cements: Unitek Multi-Cure Glass Ionomer Band Cement (3M Unitek, Monrovia, CA, USA) and Transbond Plus Light Cure Band Adhesive (3M Unitek). Appliances were subjected to MRI with two different strengths (1.5 Tesla and 3 Tesla). Tubes and band temperature was measured before and after MRI. Subsequently, the shear bond strength (SBS) test was calculated. Data underwent statistical analysis (*p* < 0.05). After MRI, molar bands exhibited significant heating, even though not clinically relevant, with a temperature increase ranging between 0.48 °C and 1.25 °C (*p* < 0.05). SBS did not show significant differences (*p* > 0.05). The present study suggests that, under MRI, the molar bands tested are safe; therefore, their removal could be not recommended for non-head and neck MRI exams. Removal would be necessary just in artifact risk areas.

## 1. Introduction

Magnetic Resonance Imaging (MRI) is an advanced diagnostic tool, applied to assess various clinical conditions [1]. It is a non-invasive technology that produces 3-dimensional anatomical images without involving the use of ionizing radiation [2] Patients undergoing fixed orthodontic treatment or retention phase may require MRI scans of head and neck region for different reasons. To date, the effects of these appliances on MRI scans are not clear. Consequently, radiologists request to remove any metal object to prevent a possible interaction during the exam [3].

In fact, all objects are magnetized when placed in a magnetic field to a degree depending on their magnetic susceptibility [4]. First, metal items produce a signal loss, defined artifact, and are visible as a black spot in the radiographic image, affecting its diagnostic capability [5]. Moreover, this interaction could displace metallic appliances, leading to injuries for patients and damaging the diagnostic imaging equipment [2]. Lastly, magnetic resonance imaging examinations induce electrical currents in metallic implants which may be capable of causing localized tissue heating [6].

Orthodontists are frequently requested to remove orthodontic appliances before performing MRI to prevent these risks, since metallic alloys of nickel, titanium and stainless steel are widely used in orthodontics [7].

On the other hand, the removal of these devices could damage the enamel structure [8], it is laborious and time-consuming both for the orthodontist and the patient. Moreover, it could lengthen the treatment time and it may lead to unwanted tooth movement [6].

Since clear guidelines are not available, orthodontists are interested in understanding the effects of orthodontic appliances on MRI to minimize the need for their removal [7,9].

At present, many studies assessed the artifacts derived from molar bands [10,11,12], but little data are available about their interaction with the magnetic field and whether they should be removed. Molar bands are used in the fixed orthodontic treatment, both with metallic and ceramic brackets [13,14]. Headgears, lingual arches, transpalatal bars or quad-helixes require molar bands to be used [15].

Therefore, the aim of this study was to evaluate the in vitro effects of MRI at different magnetic field strengths (1.5 T and 3 T) on temperature and bond strength of molar bands cemented to human teeth with two different cements. The first null hypothesis was that there was no significant difference between temperature before and after MRI. The second null hypothesis was that there was no significant difference in the Shear Bond Strength (SBS) values among different groups between temperature before and after MRI.

## 2. Materials and Methods

### 2.1. Specimen Preparation

The present in vitro study was approved by the Unit Internal Review Board (2021-0414). A total of 60 permanent human upper molars from patients aging 18–50 were collected and stored in thymol at 0.1% (*w*/*v*) according to the following criteria: intact enamel, no carious lesions, restorations or fractures [16]. In detail, they were third molars unable to erupt and periodontally compromised first/second molars. They were carefully cleaned with Softbrush Coarse 2140 burs (Edenta, Scarborough, ON, Canada) at low speed, without water spray.

Each molar was inserted inside a plastic cylindrical mold (2.0 cm height × 2.5 cm diameter) in which cold-curing fast-setting acrylic (Leocryl, Leone s.p.a., Sesto Fiorentino, Italy) was poured. Then, the mold was removed in order to cement the upper right molar bands (Calibra lower molar bands, Leone SpA). The appropriate diameter of the ring was chosen for each tooth and a palatal button and buccal double tube (Leone SpA) were welded on the band using a welding and soldering unit (Assistant 3000 110–240 V, Dentaurum GmbH & Co., Ispringen, Germany), adopting the maximum available power to avoid detachments during the SBS test. Then, teeth were divided into 6 groups of 10 specimens each, depending on the cement used and the magnetic field strength between Unitek Multi-Cure Glass Ionomer Band Cement (3M Unitek, Monrovia, CA, USA), abbreviated as RMGIC (resin-modified glass ionomer cement) and Transbond Plus Light Cure Band Adhesive (3M Unitek), abbreviated as Composite (Table 1).

Multi-Cure cement consists of two different components (powder and liquid) that require chair mixing, while Transbond Plus is sold in syringes, containing the material ready for use. Cementation was performed according to the manufacturer’s instructions and the light curing unit was performed with Starlight Pro light-emitting diode (LED) unit (Mectron SPA, Carasco, Italy), with a power output of 1400 mW/cm^2^ (Table 2).

Then, samples were stored in thymol at 0.1% (*w*/*v*).

### 2.2. Temperature Test and MRI

Specimens were left in the magnet room for 24 h before the MRI test to standardize their temperature in the testing room. A contact thermometer (PeakTech^®^ Digital Thermometer 5135/5140 Prilf—und Messtechnik GmbH, Ahrensburg, Germany) was used to measure the temperature (in Celsius degrees) of each sample. The band temperature was measured by contacting the thermometer probe at the middle height of the ring. The tube temperature was measured at the center of the mesial top border. Temperature measurements were performed immediately before (T0) and after (T1) the MRI exam.

Groups 1 and 2 did not undergo an MRI exam, while groups 3 to 6 underwent the MRI test and were carefully prepared.

Samples of every tested group were placed in a plastic box at a distance of 43 mm to simulate the intermolar width [17] and locked on a plaster base to keep them in place. Then, the plastic box was located inside the support of the patient’s head in the machine.

Groups 3 and 4 underwent magnetic resonance imaging at 1.5 T strength (model Magnetom Aera, Siemens AG, Munich, Germany), while Groups 5 and 6 underwent a 3 T MRI (Magnetom Skyra Fit model, Siemens AG, Munich, Germany).

The parameters used in MRI are provided in the Supplementary Materials (Tables S1 and S2).

The total scanning time was approximately 20 min for each group.

After MRI and temperature measurement, all the specimens were stored in thymol at 0.1% (*w*/*v*).

### 2.3. Shear Bond Strength Test (SBS)

An Instron universal testing machine (Model 3343, Instron Corp, Canton, MA, USA) was used to measure SBS. Each sample was fixed in the mechanical jaw, keeping the band wall parallel to the shearing force. The maximum load required to debond the bands was recorded in Newton (N) and converted into MegaPascals (MPa) knowing the surface area of 1.50 cm² [17]. The vertical force was set at 1 mm/min [18].

### 2.4. Statistical Analysis

Statistical analysis was performed using R software (version 3.1.3, R Development Core Team, R Foundation for Statistical Computing, Wien, Austria). Descriptive statistics, including mean, standard deviation, minimum, median and maximum values, were calculated for all groups.

The normality of distribution was calculated with the Kolmogorov and Smirnov tests. Inferential statistics were performed with the ANOVA and Tukey post hoc tests for temperatures and SBS values.

Linear regression models for tube temperature, band temperature and SBS were performed, adding as covariates the cement used and the MRI strength.

The significance was predetermined at *p* < 0.05 for all tests.

## 3. Results

### 3.1. Temperature Test

**Figure 1:**
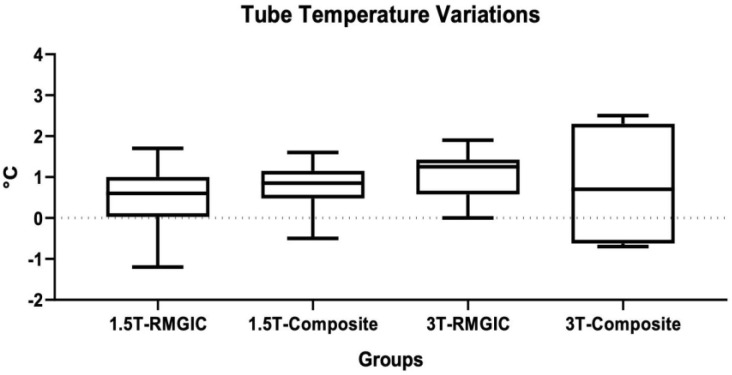
Tube temperature variation (°C) for each material before and after the 1.5 T and 3 T MRIs.

Descriptive statistics of vestibular tube temperatures recorded are shown in Table 3. ANOVA indicated the presence of significant differences between the groups (*p* < 0.0001). Considering the Tukey post hoc test, at 1.5 T no significant increase from T0 to T1 both for RMGIC and Composite materials (*p* > 0.05). At 3 T, a significant difference was found comparing RMGIC and Composite at T1 (*p* < 0.05). A significant difference was found for Composite material at T1 between 1.5 T and 3 T RMI (*p* < 0.05). Tube temperature variations are shown in Figure 1.

**Figure 2:**
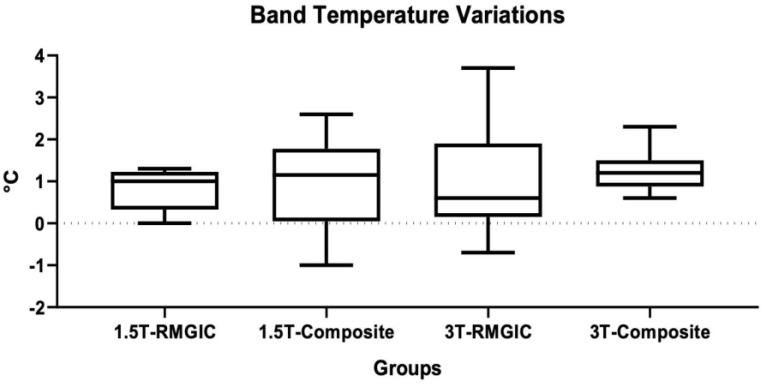
Band temperature variations (°C) for each material before and after the 1.5 T and 3 T MRIs.

Descriptive statistics of band temperatures recorded are shown in Table 4. ANOVA showed significant differences among the groups (*p* < 0.0001). Considering the Tukey post hoc test, at 1.5 T no significant differences were found for each material between T0 and T1 (*p* > 0.05). A significant difference was found between RMGIC T1 and Composite T0 (*p* < 0.05). At 3 T, significant differences were found between T0 and T1 for both materials (*p* < 0.05). No significant differences were found between the materials both at T0 and T1 (*p* > 0.05). Moreover, no significant differences were found among the groups between 1.5 T and 3 T (*p* > 0.05). Band temperature variations are shown in Figure 2.

### 3.2. SBS Test

Descriptive statistics of SBS values are reported in Table 5. ANOVA showed no significant difference in the SBS values among the various groups tested (*p* = 0.8369).

### 3.3. Linear Regressions

Linear regression models showed that tubes and band temperatures were significantly affected by the MRI strength and time frame (Table 6). On the other hand, the bonding material did not significantly influence tube and band temperatures (*p* > 0.05). SBS values were not significantly affected by the MRI strength, time or bonding material (*p* > 0.05).

## 4. Discussion

Patients wearing orthodontic devices are frequently asked to remove their appliances before an MRI exam, even when this latter regards anatomical districts far from the head and neck, in order to prevent heating and detachment of the appliances. As in other fields of dentistry, even in orthodontics, the chemical and physical characteristics of metal alloys as well as the connections between the tooth and these materials deserves to be deeply evaluated [19,20].

The first null hypothesis of this study was partially rejected. Significant differences in band and tube temperatures were reported. Linear regressions revealed significant effects of magnetic field strength and time on the temperatures of the orthodontic devices tested.

Indeed, after MRI, every group showed a temperature increase, especially after MRI at 3 T, according to the fact that heating effects are particularly relevant to high-field MRI investigations [21].

Group 6 (Composite, 3 T) revealed the highest heating of bands and tubes. These results were numerically significant, but not clinically relevant, since heating was limited to a few degrees. In fact, the temperature increase in the dental devices ranged between 0.48 °C and 1.25 °C, so it was far below the safety limit of 5.6 °C for pulpal tissue [22].

Furthermore, the heating of orthodontic devices was not clinically relevant and resulted safe for the patient or surrounding tissues [11].

Currently, many Authors evaluated artifacts derived from orthodontic devices, including molar bands [23], but only a few studies examined temperature changes after the MRI [11,20,24].

Yassi et al. [24] considered the heating of orthodontic devices after MRI at 1 T and 1.5 T and molar bands showed no significative temperature increase.

Hasegawa et al. [11] considered an orthodontic device made of orthodontic brackets, a wire and molar bands attached to a maxillary tooth model. The aim of the study was to evaluate the risk of injury during 3 T MRI. This orthodontic appliance showed heating, even though not clinically significant. Therefore, the Authors concluded that a spacer might be required between the appliance and the oral mucosa and the wire should be removed from the brackets before MRI.

In addition, Regier et al. [20] studied the effect of 3 T MRI on ten stainless steel orthodontic devices, each of them attached to molar bands. Relevant temperature increases were not observed during the exam. These results are in agreement with the present report.

Taking into account SBS, the second null hypothesis of this study was accepted. MRI Strength, bonding and time did not significantly affect SBS values. No significant differences in the adhesion values were reported among the tested groups. The Composite groups showed a slight decrease in SBS after MRI, but these values were not numerically or clinically relevant. In fact, an orthodontic biomaterial should have bonding forces between 5 and 50 MPa, to support masticatory forces without risk of enamel loss [25].

To date, no studies have evaluated the SBS of molar bands after MRI, but Sfondrini et al. [9] have recently investigated the effects of MRI on orthodontic brackets by measuring the SBS. Values obtained under all conditions tested were clinically acceptable, consequently, this is in agreement with the present report.

In addition, a previous report [24] assessed the risk of orthodontic devices displacement during MRI, considering deflection angles and translational forces evaluating many orthodontic appliances, including molar bands, to MRI at 1 T and 1.5 T. The values obtained were lower than the orthodontic and orthopedic forces. The Authors considered the risk of detachment and displacement as non-existent, the importance of following the usual recommendations and checking the proper fixation of devices prior to MRI.

When a patient with an orthodontic device requires an MRI exam, radiologists have to decide whether or not to remove it. Therefore, this report aims to give a contribution to future guidelines in order to assist radiologists and orthodontists in their work.

In the present study, molar bands did not exhibit clinically significant heating; in addition, after MRI, the adhesive forces did not change, and the devices were not displaced.

Indeed, it can be assumed that the tested devices are safe during MRI; therefore, they could not be removed prior to 1.5 T and 3 T MRI body except for head and neck imaging. On the other hand, if a head and neck MRI is required, molar bands need to be removed to avoid artifacts [7,26].

The same applies to patients undergoing multibracket therapy with molar bands: in fact, a recent study [9] showed that the removal of orthodontic brackets is not recommended for non-head and neck MRI, as temperature and SBS did not show any difference after MRI.

As regards removable devices attached to the molar bands, such as lip-bumpers or headgears, they could be easily removed by the patients prior to the MRI exam.

Appliances attached to the bands with sheets and held in place by frictional forces, as quad-helixes or palatal bars, can be removed by the orthodontist before the MRI, and then repositioned with no time-consuming procedures.

On the other hand, devices welded to molar bands such as Herbst appliances, rapid maxillary expanders, welded quad-helixes, palatal bars or lip-bumpers, cannot be removed without considerable effort, costs and interruption of orthodontic treatment. In these situations, clinicians should assess the study area, in order to decide whether to remove fixed devices and bands.

According to Regier et al. [20], who tested the heating of many orthodontic appliances such as a lip-bumper, quad-helix and a palatal expander during a 3 T MRI, a routine head examination can be safely carried out in patients. In addition, Kemper et al. [27] evaluated the translational and rotational forces of some orthodontic devices used for fixed orthodontic therapy, revealing that adequate fixation must be checked prior to MRI to minimize the possible effects of the magnetic field.

However, further studies are required to assess the potential interactions of welded orthodontic appliances with the magnetic field.

The major limitation of the present report is that it is an in vitro investigation, therefore clinical studies are necessary to confirm the finding of the study. Additionally, the results here obtained are valid only for the materials tested, as there may be variations in the induced magnetic moments among devices of different manufacturers. Moreover, since 6 T MRI appliances are available, future studies are recommended to analyze possible different interactions between orthodontic appliances and the higher magnetic field. Investigating the possible effects of RMI on a full arch with brackets, archwires and tubes could probably complete the results here obtained. At last, an interesting aspect could be investigating patients’ perspective during MRI while they are wearing orthodontic devices.

## 5. Conclusions

In conclusion, the present report suggests that molar bands are safe as the tested materials did not exhibit clinically significant heating (from 0.48 °C to 1.25 °C), so the magnetic field did not affect temperature or adhesion in any group, and it did not cause their displacement. Therefore, the removal could not be recommended routinely but just in case of artifact risk which could compromise the diagnostic quality of the exam.

## Figures and Tables

**Table 1:** Groups tested in the present study. *: RMGIC = Unitek Multi-Cure Glass Ionomer Band Cement; Composite = Transbond Plus Light Cure Band Adhesive.

Group	MRI	Cement *
1	No MRI	RMGIC
2	No MRI	Composite
3	1.5 T MRI	RMGIC
4	1.5 T MRI	Composite
5	3 T MRI	RMGIC
6	3 T MRI	Composite

**Table 2:** Protocols used to cement bands.

Multi-Cure Cement	Transbond Plus Cement
Tooth was washed and dried with an oil-free air steam	Tooth was washed and dried with an oil-free air steam
2. Liquid and powder components were mixed using a spatula on plasticized paper according to the manufacturer instructions	2. Cement was applied to the band from the syringe 3. Band was localized on the molar
3. Cement was applied on the band 4. Band was localized on the molar	4. A probe was used to remove the excess material
5. Excess of the material was removed with a probe 6. Cement was cured with a curing light at a distance of 1–2 mm for 20 s on the occlusal surface	5. Cement was cured with a curing light at a distance of 1–2 mm for 20 s on the occlusal surface
7. Waiting for 5 min to complete the chemical hardening	

**Table 3:** Descriptive statistics tube temperatures (°C) in the various groups tested (T0: before MRI and T1: after MRI). * denotes Tukey grouping: means with the same letters are not significantly different.

Magnetic Field Strength	Bonding	Time	Mean	SD	Min	Mdn	Max	Significance *
1.5 T	RMGIC	T0	22.69	1.00	21.6	22.25	24.8	A,C,D
1.5 T	RMGIC	T1	23.17	0.34	22.7	23.3	23.6	A,B,C,D,E
1.5 T	Composite	T0	22.24	0.86	20.9	22	23.9	A,B
1.5 T	Composite	T1	23.02	0.48	22.5	22.9	24	A,B,C
3 T	RMGIC	T0	23.11	0.86	22.2	23	24.7	A,B,C,D,E
3 T	RMGIC	T1	24.19	0.60	23.4	24	25.2	E,F
3 T	Composite	T0	22.71	1.40	20.9	23.3	24.4	A,C,D,E
3 T	Composite	T1	23.53	0.45	23.2	23.4	24.7	D

**Table 4:** Descriptive statistics of band temperatures (°C) in the various groups tested (T0: before MRI and T1: after MRI). * denotes Tukey grouping means with the same letters are not significantly different.

Magnetic Field Strength	Bonding	Time	Mean	SD	Min	Mdn	Max	Significance *
1.5 T	RMGIC	T0	21.95	0.48	21.5	21.9	23	A,B
1.5 T	RMGIC	T1	22.74	0.62	21.9	22.9	23.5	B,C,D
1.5 T	Composite	T0	21.75	0.22	21.3	21.75	22.1	A
1.5 T	Composite	T1	22.69	1.10	21	22.9	24.3	A,C,D
3 T	RMGIC	T0	22.53	0.34	22.2	22.5	23.4	A,C
3 T	RMGIC	T1	23.59	1.33	22.4	23	26	D,E
3 T	Composite	T0	21.99	0.31	21.4	22.1	22.5	A,B
3 T	Composite	T1	23.24	0.30	23	23.15	24	C,D

**Table 5:** Descriptive statistics of SBS values (MPa) tested at different magnetic field strengths. * denotes Tukey grouping: means with the same letters are not significantly different.

Group	Magnetic Field Strength	Bonding	Mean	SD	Min	Mdn	Max	Significance *
1	No MRI	RMGIC	1.19	0.31	0.94	1.11	2.02	A
2	No MRI	Composite	1.39	0.37	0.68	1.39	1.91	A
3	1.5 T	RMGIC	1.21	0.52	0.49	1.17	2.18	A
4	1.5 T	Composite	1.23	0.21	0.93	1.21	1.63	A
5	3 T	RMGIC	1.31	0.31	0.83	1.26	1.88	A
6	3 T	Composite	1.27	0.39	0.75	1.23	1.91	A

**Table 6:** Results of the linear regression models of the different variables (band and tube temperature). Predictors were bonding, time and magnetic field strength. * denotes significant regressions. ns: not significant.

Dependent Variable	Independent Variable	*p* Value
Tube temperature	Strength	0.0041 *
	Time	0.0001 *
	Bonding	ns
Band temperature	Strength	0.0054 *
	Time	<0.0001 *
	Bonding	ns

## Data Availability

Data are available upon motivated request to the corresponding authors.

## Supplementary Materials

The following supporting information can be downloaded at: https://www.mdpi.com/article/10.3390/ma16020651/s1, Table S1: Parameters of 1.5 T RMI; Table S2: Parameters of 3 T RMI.
