# Supplementary material for: Effect of Magnetic Resonance Imaging at 1.5 T and 3 T on Temperature and Bond Strength of Orthodontic Bands with Welded Tubes: An In Vitro Study
Source: Materials (Basel). 2023 Jan 9;16(2):651. doi: 10.3390/ma16020651 (PMC9863444; doi:10.3390/ma16020651)
Supplement: Supplementary file 1 [file materials-16-00651-s001.zip › materials-2097008-supplementary.pdf]

materials

Supplementary Materials

Effect of Magnetic Resonance Imaging at 1.5 T and 3 T on Temperature and Bond Strength of Orthodontic Bands with Welded Tubes: An In Vitro Study

Maria Francesca Sfondrini [1], Simone Gallo [1,*], Maurizio Pascadopoli [1,*], Cinzia Rizzi [1], Andrea Boldrini [1], Simone Santagostini [2], Luca Anemoni [3], Maria Sole Prevedoni Gorone [2], Lorenzo Preda [2,4], Paola Gandini [1] and Andrea Scribante [1]

Table S1. Parameters of 1.5T RMI.

	T2-TSE Axial	T1-TSE Axial	T2-TSE Coronal	T2-FL2D Axial	EP2D 7B Axial	T2-FLAIR Axial	T1-VIBE Axial
FOV (mm)	240	240	180	210	340	240	240
Voxel size (mm)	0.5 × 0.5 × 3.0	0.5 × 0.5 × 3.0	0.6 × 0.6 × 2.0	0.5 × 0.5 × 3.0	1.1 × 1.1 × 4.0	0.5 × 0.5 × 3.0	0.6 × 0.6 × 0.6
Slice Thickness	3.0	3.0	2.0	3.0	4.0	3.0	0.6
Slices	52	52	39	45	20	20	/
TE (ms)	108	8.6	79	25	81	94	2.46
TR (ms)	5410	739	7630	1440	4900	4860	5.35
Scan time (min:s)	03:27	02:16	02:26	06:29	04:34	07:27	04:32
SAR whole body (W/kg)	1	1	1	1	1	1	1

Legend: T2 weighted turbo spin echo in axial projection (T2W-TSE AXIAL), T2 weighted turbo spin echo in coronal projection (T2W-TSE CORONAL), T2 fluid attenuated inversion recovery in axial projection (T2-FLAIR AXIAL), and T1 volumetric interpolated breath-hold examination in three dimensions fat saturated (T1 VIBE 3D FS); field of view (FOV), time of echo (TE), repetition time (TR), and specific absorption rate (SAR) of the whole body.

Table S2. Parameters of 3T RMI.

	T2-TSE Axial	T1-TSE Axial	T2-TSE Coronal	T2-FL2D HEMO Axial	EP2D DIFF 7B VALUE Axial	T2-FLAIR Axial	T1-VIBE 3D FS Axial
FOV (mm)	240	240	180	240	230	240	240
Voxel size (mm)	0.5 × 0.5 × 3.0	0.3 × 0.3 × 3.0	0.6 × 0.6 × 2.0	0.5 × 0.5 × 3.0	1.1 × 1.1 × 4.0	0.5 × 0.5 × 3.0	0.6 × 0.6 × 0.6
Slice Thickness	3.0	3.0	2.0	3.0	4.0	3.0	0.6
Slices	52	52	39	45	20	40	/
TE (ms)	104	10	75	12	58	90	2.48
TR (ms)	6260	689	7630	801	3300	8000	5.30
TIR (ms)	/	/	/	/	/	2368	
Scan time (min:s)	03:09	02:52	02:26	03:11	03:02	02:56	04:26
SAR whole body (W/kg)	1	1	0.9	0.9	0.9	0.8	0.7

Legend: T2 weighted two-dimensional fast low angle shot for hemosiderin detection in axial projection (T2W-FL2D HEMO AXIAL), T2 weighted turbo inversion recovery magnitude in axial projection (T2W-TIRM AXIAL), two-dimensional echo planar with 5 mm slice diffusion in axial projection (EP2D DIFF 5 mm AXIAL), 2 dimensional echo planar with 3 mm slice diffusion in axial projection (EP2D DIFF 3 mm AXIAL), and proton density weighted in axial projection (PDw AXIAL); field of view (FOV), time of echo (TE), repetition time (TR), turbo inversion recovery (TIR), and specific absorption rate (SAR) of the whole body.
